# Machine learning-based predictive tools and nomogram for in-hospital mortality in critically ill cancer patients: development and external validation using retrospective cohorts

**DOI:** 10.1186/s12911-025-03054-z

**Published:** 2025-07-04

**Authors:** Kaier Gu, Saisai Lu

**Affiliations:** 1 Department of Internal Medicine, Shaoxing Maternity and Child Health Care Hospital, Shaoxing, Zhejiang China; 2 Department of Rheumatology, The First Affiliated Hospital of Wenzhou Medical University, Wenzhou, Zhejiang China

**Keywords:** Malignancy, Cancer, Intensive care unit, Machine learning, In-hospital mortality, Prognosis

## Abstract

**Background:**

The incidence of intensive care unit (ICU) admissions and the corresponding mortality rates among cancer patients are both high. However, the existing scoring systems all lack specificity. This research seeks to establish and validate a prediction model for early forecasting of in-hospital mortality in critically ill cancer patients.

**Methods:**

A retrospective analysis was conducted utilizing data from cancer patients obtained from the eICU and MIMIC-IV databases. The least absolute shrinkage and selection operator method was employed to screen predictive factors and construct six machine learning (ML) models. These models were mainly compared in terms of their predictive performance through area under the curve (AUC) and underwent external validation. Nomograms were developed using multivariate logistic regression (LR) analysis findings. The Shapley Additive exPlanations (SHAP) method was employed to explain the variables within the ML models.

**Results:**

Twelve predictive factors were chosen to develop the ML models. Among these models, the LR model and the eXtreme gradient boosting (XGB) model demonstrated the optimal efficacy. In the external validation cohort, their AUC values reached 0.751 [95% confidence interval (CI): 0.735 − 0.768] and 0.737 (95% CI: 0.720 − 0.754), respectively. Moreover, nomograms and SHAP were employed to explain the variables. Additionally, a user-friendly web-based calculator tool was created.

**Conclusions:**

The LR and XGB models were successfully developed to predict in-hospital mortality in critically ill cancer patients. Their robust predictive ability was demonstrated in the external validation cohorts. This model can assist physicians in clinical decision-making and timely intervention.

**Clinical trial number:**

Not applicable.

**Supplementary information:**

The online version contains supplementary material available at 10.1186/s12911-025-03054-z.

## Background

Cancer has consistently posed a significant challenge to global public health [1]. With the advancements in the field of oncology, the survival period of cancer patients has witnessed a notable extension. Nevertheless, the proportion of cancer patients being transferred to the intensive care unit (ICU) remains substantial [2]. Research indicates that approximately 5% of cancer patients require admission to the ICU due to critical conditions within two years of diagnosis, and approximately 15% of patients in the ICU have cancer [3–5]. Hence, accurately evaluating the prognosis of these critically ill cancer patients is essential for developing personalized treatment plans and improving patient survival rates.

While a range of clinical scoring systems, including APACHE II, SAPS II, and SOFA, are widely used in ICUs to evaluate patient mortality risk, these models are predominantly designed for the general critically ill population and do not adequately account for the unique pathological mechanisms associated with cancer [6–10]. Consequently, existing tools exhibit insufficient predictive efficacy for ICU cancer patients, creating a situation where clinicians are “data-rich but insight-poor” when making clinical decisions.

In recent years, machine learning (ML) technology has exhibited tremendous potential in the domain of medical prediction owing to its capacity to handle high-dimensional and nonlinear data. ML models can integrate multimodal data, such as electronic health records, radiomics, and biomarkers, to uncover latent risk patterns and have achieved breakthroughs in scenarios like prognosis prediction of sepsis and prediction of cardiac arrest [11, 12]. To date, a considerable body of studies have explored the application of ML models in predicting the prognosis of patients with various types of cancers, such as lung cancer and colorectal cancer [13–16]. However, for all cancer patients, particularly those in the ICU, current research remains conspicuously inadequate. Currently, there is no interpretable ML model specifically dedicated to predicting the in-hospital mortality of all cancer patients in the ICU.

This study aims to fill this void by constructing a prediction model for in-hospital mortality specifically designed for critically ill cancer patients through ML. Furthermore, the performance of the model will be further evaluated through an external validation cohort to ensure its reliability and generalization ability. We anticipate that the outcomes of this study will furnish a more precise and dynamic risk assessment tool for the field of critical oncology, thereby facilitating clinicians in formulating timely and effective treatment strategies.

## Methods

### Data sources

The primary data sources employed in this study are two databases, namely the eICU database and the MIMIC-IV database [17, 18]. The training cohort and internal validation cohort data utilized in the construction of the ML model were retrieved from the eICU database.

The eICU database encompasses inpatient information of ICU patients from 208 hospitals in the United States. To protect patient privacy, all patient personal data have been anonymized, and identities have been replaced with random numbers.

The external validation cohort originated from the MIMIC-IV database. The MIMIC-IV database is a multi-parametric intensive care database made publicly accessible by the MIT Laboratory for Computational Physiology (https://physionet.org). The MIMIC-IV database is also an anonymized multi-center database.

### Inclusion and exclusion criteria

**Fig. 1:**
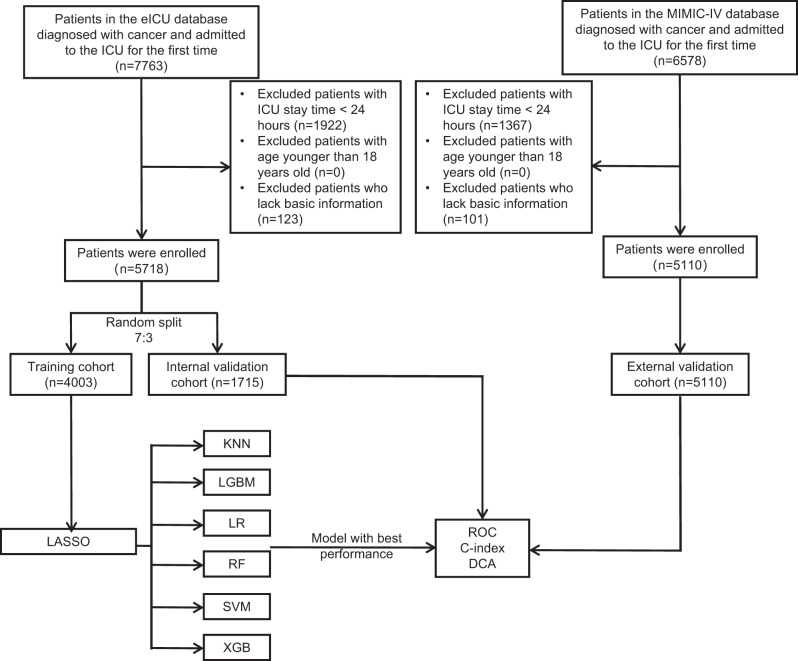
Flow chart of the study. ICU: Intensive care unit; LASSO: Least absolute shrinkage and selection operator; KNN: K-nearest neighbor; LGBM: Light gradient boosting machine; LR: Logistic regression; RF: Random forest; SVM: Support vector machine; XGB: eXtreme gradient boosting; ROC: Receiver operating characteristic; DCA: Decision curve analysis

Patients with the admission diagnosis of “cancer” and their first ICU admission were separately extracted from the databases.Cancer was defined in accordance with the International Classification of Diseases, Ninth Revision (ICD-9) and Tenth Revision (ICD-10) codes. See Table S1 for details of codes used to identify each cancer patient in ICD-9 and ICD-10. Exclusion Criteria: (1) ICU length of stay < 24 hours; (2) Age < 18 years old; (3) Basic information of the patient was missing. The screening process is depicted as shown in Fig. 1.

### Data collection and processing

Data from the first 24 hours following ICU admission were compiled for each patient. The potential variables of the prediction model were collected: demographic characteristics, comorbidities, laboratory indicators, vital signs, intervention measures, and outcomes. Since the data originated from two different databases, the same variables were extracted as far as possible.

Variables with a missing value percentage exceeding 20% were excluded (Table S2). For variables with a missing value percentage ranging from 10% to 20%, multiple imputation was employed to address the issue of missing data. For categorical variables with a missing value percentage of less than 10%, the mode was utilized for imputation. For continuous variables with a missing value percentage of less than 10%, the median was utilized for imputation.

Ultimately, the following demographic characteristics were retrieved: age and gender. The comorbidities encompassed hypertension, diabetes, liver disease, peripheral vascular disease, cerebrovascular disease, heart failure, atrial fibrillation, coronary artery disease, chronic renal disease, acute kidney injury (AKI), and sepsis. The maximum values recorded within 24 hours after admission were used for the following variables: white blood cell, potassium, chloride, glucose, creatinine, blood urea nitrogen (BUN), temperature, heart rate (HR), and respiratory rate (RR). The minimum values recorded within 24 hours after admission were used for the following variables: hemoglobin (Hb), platelet, sodium, calcium, bicarbonate, systolic blood pressure (SBP), diastolic blood pressure, and peripheral oxygen saturation (SpO_2_). The intervention measures encompassed mechanical ventilation (MV), continuous renal replacement therapy (CRRT), vasopressor, antibiotic, antiarrhythmic, blood product, diuretic, and sedative.

### Outcome measures

The principal clinical endpoint of this investigation was in-hospital mortality occurring subsequent to the initial 24-hour ICU admission period. Additional endpoints comprised ICU mortality rate, duration of hospitalization, and critical care unit stay duration.

### Model construction and validation

The eICU database population underwent randomized allocation into training and internal validation cohorts through a ratio of 7:3, while the MIMIC-IV dataset served as an independent external validation cohort. The training cohort was utilized to construct the models of ML models, while the internal and external validation cohorts were employed to assess the performance of these models. Owing to the numerous variables, least absolute shrinkage and selection operator (LASSO) regression analysis was adopted to obtain the variables closely related to the outcome. Ten-fold cross-validation was conducted to mitigate overfitting and enhance model stability. The linear correlation among variables was quantitatively examined using the Variance Inflation Factor. The ultimately selected variables were input into six ML models, including k-nearest neighbor (KNN), light gradient boosting machine (LGBM), logistic regression (LR), random forest (RF), support vector machine (SVM), and eXtreme gradient boosting (XGB), for model construction. The discriminatory performance of the models was primarily evaluated using the receiver operating characteristic (ROC) curve and the area under the curve (AUC). Internal validation was conducted through 1,000 Bootstrap resampling iterations to correct for overfitting bias and calculate the 95% confidence interval. Furthermore, various performance metrics, including sensitivity, specificity, positive predictive value (PPV), negative predictive value, and F1 score, were evaluated. The Hosmer-Lemeshow test and calibration curves were utilized to appraise the calibration of the models. The clinical effectiveness of the models was additionally assessed through calibration plots and decision curve analysis (DCA). The Shapley Additive exPlanations (SHAP) method was employed to explain the variables within the ML models.

### Statistical analysis

The statistical analyses and model construction and validation were carried out via the R software package (version 4.2.1) and DCPM (V4.01, Jingding Medical Technology Co., Ltd.). The normality of continuous variables was assessed through the Kolmogorov-Smirnov test. As all continuous variables were non-normal, they were depicted by the median (interquartile range), and the Mann-Whitney U test was employed to compare differences between groups. Categorical variables were presented as percentages (%) and compared between groups using the chi-square test or Fisher’s exact test as appropriate. A P-value < 0.05 was regarded as statistically significant.

## Results

### Patient characteristics

Table 1 presents a comparison of baseline characteristics of critically ill cancer in the eICU and MIMIC-IV databases. The results show that the overall condition of patients in MIMIC-IV was more severe, with an in-hospital mortality of 20.0%, significantly higher than that in eICU (14.7%).

**Table 1:** Baseline characteristics of critically ill cancer patients in the eICU and MIMIC-IV databases

Characteristics	eICU				MIMIC-IV				The P-value between the two databases
	Total (n = 5718)	Survival group (n = 4879)	Death group (n = 839)	P-value	Total (n = 5110)	Survival group (n = 4088)	Death group (n = 1022)	P-value	
**Demographic characteristics**									
Age, years	67 [58, 76]	67 [58, 75]	68 [59, 76]	0.047	69 [60, 78]	69 [60, 78]	70 [61, 79]	0.054	<0.001
Gender, male, %	3119 (54.6%)	2660 (54.2%)	459 (54.7%)	0.949	3001 (58.7%)	2442 (59.7%)	559 (54.7%)	0.004	<0.001
**Comorbidities**									
Hypertension, %	932 (16.3%)	808 (16.6%)	124 (14.8%)	0.215	2146 (42.0%)	1750 (42.8%)	396 (38.8%)	0.020	<0.001
Diabetes, %	119 (2.1%)	93 (1.9%)	26 (3.1%)	0.035	1245 (24.4%)	980 (24.0%)	265 (25.9%)	0.207	<0.001
Liver disease, %	251 (4.4%)	162 (3.3%)	89 (10.6%)	<0.001	1302 (25.5%)	890 (21.8%)	412 (40.3%)	<0.001	<0.001
Peripheral vascular disease, %	226 (4.0%)	181 (3.7%)	45 (5.4%)	0.030	134 (2.6%)	100 (2.5%)	34 (3.3%)	0.143	<0.001
Cerebrovascular disease, %	181 (3.2%)	146 (3.0%)	35 (4.2%)	0.090	619 (12.1%)	474 (11.6%)	145 (14.2%)	0.027	<0.001
HF, %	426 (7.5%)	337 (6.9%)	89 (10.6%)	<0.001	981 (19.2%)	752 (18.4%)	229 (22.4%)	0.004	<0.001
AF, %	651 (11.4%)	503 (10.3%)	148 (17.6%)	<0.001	1347 (26.4%)	1031 (25.2%)	316 (30.9%)	<0.001	<0.001
CAD, %	278 (4.9%)	233 (4.8%)	45 (5.4%)	0.519	920 (18.0%)	739 (18.1%)	181 (17.7%)	0.820	<0.001
CKD, %	474 (8.3%)	387 (7.9%)	87 (10.4%)	0.022	814 (15.9%)	636 (15.6%)	178 (17.4%)	0.160	<0.001
AKI, %	805 (14.1%)	556 (11.4%)	249 (29.7%)	<0.001	2818 (55.2%)	2078 (50.8%)	740 (72.4%)	<0.001	<0.001
Sepsis, %	1210 (21.2%)	873 (17.9%)	337 (40.2%)	<0.001	2492 (48.8%)	1803 (44.1%)	689 (67.4%)	<0.001	<0.001
**Laboratory indicators**									
WBC_max, 10^9^/L	11.1 [7.6, 15.2]	11.0 [7.7, 14.9]	11.9 [6.7, 18.3]	0.008	11.6 [7.8, 16.5]	11.3 [7.7, 15.8]	13.0 [8.3, 19.1]	<0.001	<0.001
Hb_min, g/dL	10.1 [8.73, 11.5]	10.2 [8.8, 11.6]	9.4 [8.3, 10.9]	<0.001	9.6 [8.2, 11.3]	9.8 [8.4, 11.4]	9.1 [7.8, 10.7]	<0.001	<0.001
PLT_min, 10^9^/L	187 [129, 256]	190 [136, 256]	165 [72, 254.50]	<0.001	191 [123, 271]	194 [132, 270]	170 [88, 278]	<0.001	0.064
Potassium_max, mmol/L	4.10 [3.80, 4.50]	4.10 [3.80, 4.50]	4.25 [3.80, 4.80]	<0.001	4.30 [4.00, 4.70]	4.30 [3.90, 4.70]	4.50 [4.00, 5.10]	<0.001	<0.001
Sodium_min, mmol/L	138 [135, 140]	137 [135, 140]	138 [134, 141]	0.904	137 [134, 140]	137 [135, 140]	136 [133, 140]	<0.001	<0.001
Chloride_max, mmol/L	105 [101, 108]	105 [101, 108]	104 [100, 109]	0.040	104 [101, 108]	105 [101, 108]	104 [99, 108]	0.001	0.487
Calcium_min, mg/dL	8.1 [7.6, 8.6]	8.1 [7.7, 8.6]	8.0 [7.4, 8.7]	<0.001	8.2 [7.6, 8.7]	8.2 [7.7, 8.7]	8.0 [7.4, 8.6]	<0.001	0.151
Glucose_max, mg/dL	138 [114, 168]	138 [114, 168]	135 [110, 170]	0.133	136 [112, 174]	135 [113, 170]	141 [111, 187]	0.015	0.421
Cr_max, umol/L	0.92 [0.69, 1.38]	0.90 [0.68, 1.30]	1.21 [0.80, 2.06]	<0.001	1.00 [0.70, 1.50]	0.90 [0.70, 1.30]	1.20 [0.80, 2.00]	<0.001	0.001
BUN_max, mmol/L	18.0 [12.0, 29.0]	16.5 [11.7, 26.0]	29.0 [17.0, 45.5]	<0.001	21.0 [14.0, 32.0]	19.0 [14.0, 29.0]	29.0 [19.0, 48.0]	<0.001	<0.001
Bicarbonate_min, mmol/L	24.0 [21.0, 26.3]	24.0 [21.3, 26.5]	22.0 [18.5, 26.0]	<0.001	22.0 [19.0, 25.0]	23.0 [20.0, 25.0]	21.0 [17.0, 24.0]	<0.001	<0.001
**Vital signs**									
T_max, °C	36.9 [36.6, 37.2]	36.9 [36.7, 37.2]	36.9 [36.6, 37.4]	0.703	37.2 [36.9, 37.6]	37.2 [36.9, 37.6]	37.2 [36.8, 37.7]	0.395	<0.001
HR_max, bpm	94 [81, 108]	92 [80, 106]	106 [93, 120]	<0.001	107 [93, 122]	104 [91, 119]	117 [101, 134]	<0.001	<0.001
RR_max, bpm	20 [16, 18]	20 [16, 19]	24 [20, 21]	<0.001	28 [18, 22]	27 [18, 22]	31 [23, 24]	<0.001	<0.001
SBP_min, mmHg	110 [99, 125]	112 [100, 126]	103 [92, 117]	<0.001	91 [82, 104]	93 [83, 105]	86 [76, 96]	<0.001	<0.001
DBP_min, mmHg	60 [53, 68]	61 [53, 68]	58 [50, 66]	<0.001	48 [41, 56]	49 [42, 57]	46 [38, 54]	<0.001	<0.001
SpO_2__min, %	97 [95, 98]	97 [95, 98]	96 [93, 98]	<0.001	92 [90, 94]	92 [90, 94]	91 [87, 94]	<0.001	<0.001
**Intervention measures**									
MV, %	309 (6.0%)	263 (6.0%)	46 (6.2%)	0.930	2977 (58.3%)	2443 (59.8%)	534 (52.3%)	<0.001	<0.001
CRRT, %	180 (3.2%)	104 (2.1%)	76 (9.1%)	<0.001	46 (0.9%)	21 (0.5%)	25 (2.5%)	<0.001	<0.001
Vasopressor, %	844 (14.8%)	591 (12.1%)	253 (30.2%)	<0.001	1564 (30.6%)	1112 (27.0%)	452 (44.2%)	<0.001	<0.001
Antibiotic, %	717 (12.5%)	579 (11.9%)	138 (16.5%)	<0.001	3155 (61.7%)	2377 (58.2%)	778 (76.1%)	<0.001	<0.001
Antiarrhythmic, %	564 (9.9%)	480 (9.8%)	84 (10.0%)	0.926	1351 (26.4%)	1086 (26.6%)	265 (25.9%)	0.709	<0.001
Diuretic, %	410 (7.2%)	328 (6.7%)	82 (9.8%)	0.002	862 (16.9%)	633 (15.5%)	229 (22.4%)	<0.001	<0.001
Blood product, %	280 (4.9%)	222 (4.6%)	58 (6.9%)	0.004	1555 (30.4%)	1215 (29.7%)	340 (33.3%)	0.030	<0.001
Sedative, %	1176 (20.6%)	971 (19.9%)	205 (24.4%)	0.003	4352 (85.2%)	3340 (81.7%)	1012 (99.0%)	<0.001	<0.001
**Outcomes**									
In-hospital mortality, %	839 (14.7%)	0 (0.00%)	839 (100%)	-	1022 (20.0%)	0 (0.00%)	1022 (100%)	-	<0.001
ICU mortality, %	465 (8.1%)	0 (0.00%)	465 (54.4%)	-	634 (12.41%)	0 (0.00%)	634 (62.0%)	-	<0.001
Hospital length of stay, days	7.9 [5.0, 12.9]	8.0 [5.1, 12.8]	7.3 [3.9, 14.1]	0.002	8.7 [5.3, 14.7]	8.7 [5.6, 14.6]	8.4 [4.1, 15.2]	0.002	<0.001
ICU length of stay, days	2.6 [1.7, 4.2]	2.5 [1.7, 4.0]	3.4 [2.0, 6.1]	<0.001	2.5 [1.7, 4.2]	2.3 [1.6, 3.9]	3.4 [2.0, 6.6]	<0.001	0.363

HF: Heart failure; AF: Atrial fibrillation; CAD: Coronary artery disease; CKD: Chronic kidney disease; AKI: Acute kidney injury; WBC: White blood cell; Hb: Hemoglobin; PLT: Platelet; Cr: Creatinine; BUN: Blood urea nitrogen; T: Temperature; HR: Heart rate; RR: Respiratory rate; SBP: Systolic blood pressure; DBP: Diastolic blood pressure; SpO_2_: Peripheral oxygen saturation; MV: Mechanical ventilation; CRRT: Continuous renal replacement therapy; ICU: Intensive care unit

Patients in the death group showed significant differences from those in the survival group in multiple aspects: In terms of comorbidities, the incidence rates of liver disease, AKI, and sepsis were significantly higher (all P < 0.001). Laboratory indicators and vital signs revealed that the levels of potassium, creatinine, urea nitrogen, HR, and RR were significantly higher in the death group than in the survival group, while Hb, platelet, calcium, bicarbonate levels, SBP, diastolic blood pressure, and SpO_2_ were significantly lower (all P < 0.001). Regarding intervention measures, the death group received CRRT, vasopressor, and antibiotic more frequently (all P < 0.001). Furthermore, the ICU length of stay of the death group was significantly longer (P < 0.001). Overall, death patients exhibited more severe pathophysiological disorders, higher intensity of medical intervention needs, and poorer clinical outcomes.

### Selection of model predictors

**Fig. 2:**
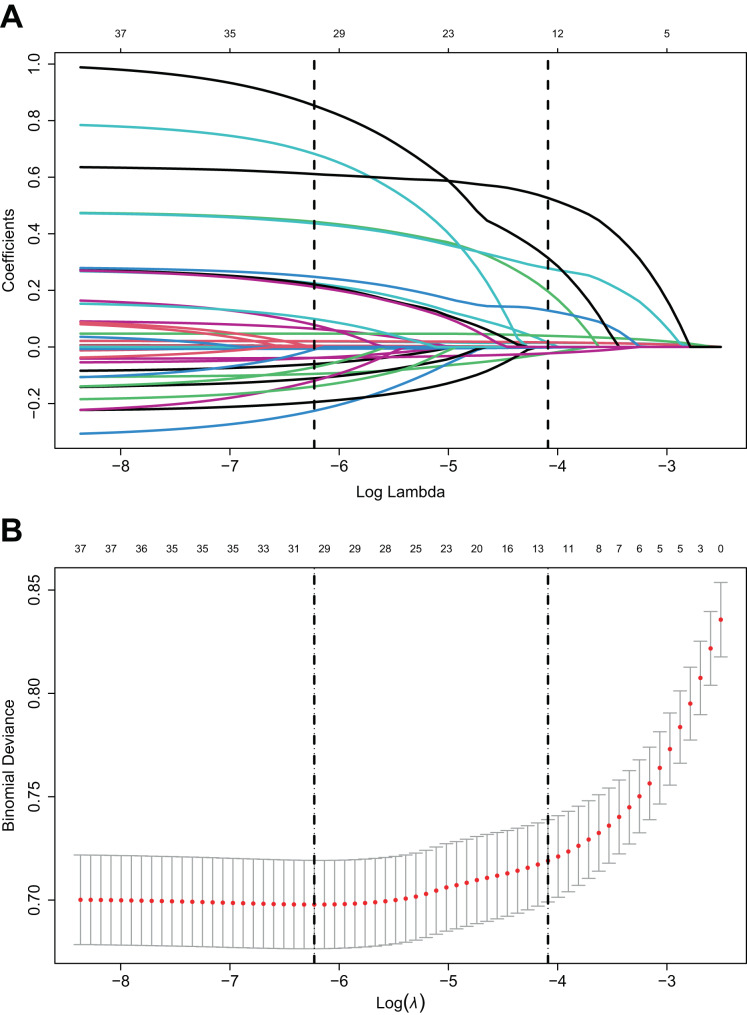
Selection of clinical variables using LASSO regression. (**A**) The coefficient values of variables in relation to log(λ); (**B**) The left vertical line demarcates the position of the minimum cross-validation error occurs, while the right vertical line demarcates the position of the minimum cross-validation error plus one standard deviation. LASSO: Least absolute shrinkage and selection operator

Statistical analysis revealed no significant differences between training cohort and an internal validation cohort (Table 2). LASSO was employed to screen the relevant variables in the training cohort (Fig. 2 and Table S3). When the λ value was 0.001973207, the minimum cross-validation error was determined, and thirty clinical variables with non-zero coefficients were obtained. When the λ value was 0.0167674, the minimum cross-validation error plus one standard deviation was determined, and twelve clinical variables with non-zero coefficients were obtained (Hb_min, Potassium_max, BUN_max, HR_max, RR_max, SBP_min, SpO_2__min, Liver_disease, AKI, Sepsis, CRRT, Vasopressor).

**Table 2:** Baseline comparison of patients with training cohort and internal validation cohort

**Characteristics**	**Total (n = 5718)**	**Training cohort (n = 4003)**	**Internal validation cohort (n = 1715)**	**P-value**
**Demographic characteristics**				
Age, years	67 [58, 76]	67 [58, 75]	67 [58, 76]	0.413
Gender, male, %	3119 (54.6%)	2171 (54.2%)	948 (55.3%)	0.486
**Comorbidities**				
Hypertension, %	932 (16.3%)	665 (16.6%)	267 (15.6%)	0.347
Diabetes, %	119 (2.1%)	88 (2.2%)	31 (1.8%)	0.397
Liver disease, %	251 (4.4%)	177 (4.4%)	74 (4.3%)	0.912
Peripheral vascular disease, %	226 (4.0%)	166 (4.2%)	60 (3.5%)	0.281
Cerebrovascular disease, %	181 (3.2%)	133 (3.3%)	48 (2.8%)	0.340
HF, %	426 (7.5%)	289 (7.2%)	137 (8.0%)	0.337
AF, %	651 (11.4%)	459 (11.5%)	192 (11.2%)	0.802
CAD, %	278 (4.9%)	198 (5.0%)	80 (4.7%)	0.699
CKD, %	474 (8.3%)	341 (8.5%)	133 (7.8%)	0.364
AKI, %	805 (14.1%)	571 (14.3%)	234 (13.6%)	0.564
Sepsis, %	1210 (21.2%)	838 (20.9%)	372 (21.7%)	0.544
**Laboratory indicators**				
WBC_max, 10^9^/L	11.1 [7.6, 15.2]	11.1 [7.7, 15.1]	11.1 [7.8, 15.1]	0.689
Hb_min, g/dL	10.1 [8.73, 11.5]	10.1 [8.7, 11.5]	10.1 [8.8, 11.6]	0.143
PLT_min, 10^9^/L	187 [129, 256]	187 [132, 251]	187 [133, 253]	0.402
Potassium_max, mmol/L	4.10 [3.80, 4.50]	4.10 [3.80, 4.50]	4.11 [3.83, 4.50]	0.106
Sodium_min, mmol/L	138 [135, 140]	138 [135, 140]	137 [135, 140]	0.233
Chloride_max, mmol/L	105 [101, 108]	105 [101, 108]	105 [101, 108]	0.799
Calcium_min, mg/dL	8.1 [7.6, 8.6]	8.1 [7.7, 8.6]	8.1 [7.7, 8.6]	0.607
Glucose_max, mg/dL	138 [114, 168]	138 [114, 167]	138 [114, 167]	0.771
Cr_max, umol/L	0.92 [0.69, 1.38]	0.92 [0.69, 1.35]	0.92 [0.70, 1.38]	0.693
BUN_max, mmol/L	18.0 [12.0, 29.0]	18.0 [12.0, 29.0]	17.5 [12.0, 28.0]	0.498
Bicarbonate_min, mmol/L	24.0 [21.0, 26.3]	24.0 [21.0, 26.0]	24.0 [21.3, 26.0]	0.528
**Vital signs**				
T_max, °C	36.9 [36.6, 37.2]	36.9 [36.7, 37.2]	36.9 [36.6, 37.2]	0.663
HR_max, bpm	94 [81, 108]	94 [82, 108]	94 [82, 107]	0.506
RR_max, bpm	20 [16, 18]	20 [17, 18]	20 [16, 18]	0.351
SBP_min, mmHg	110 [99, 125]	110 [100, 124]	110 [99, 125]	0.648
DBP_min, mmHg	60 [53, 68]	60 [53, 67]	60 [54, 68]	0.354
SpO_2__min, %	97 [95, 98]	96 [95, 98]	97 [95, 98]	0.561
**Intervention measures**				
MV, %	309 (6.0%)	218 (5.5%)	91 (5.3%)	0.880
CRRT, %	180 (3.2%)	116 (2.9%)	64 (3.7%)	0.116
Vasopressor, %	844 (14.8%)	587 (14.7%)	257 (15.0%)	0.785
Antibiotic, %	717 (12.5%)	518 (12.9%)	199 (11.6%)	0.175
Antiarrhythmic, %	564 (9.9%)	379 (9.5%)	185 (10.8%)	0.138
Diuretic, %	410 (7.2%)	287 (7.2%)	123 (7.7%)	1.000
Blood product, %	280 (4.9%)	193 (4.8%)	87 (5.1%)	0.736
Sedative, %	1176 (20.6%)	829 (20.7%)	347 (20.2%)	0.709
**Outcomes**				
In-hospital mortality, %	839 (14.7%)	591 (14.8%)	248 (14.5%)	0.798
ICU mortality, %	465 (8.1%)	313 (7.8%)	152 (8.9%)	0.204
Hospital length of stay, days	7.9 [5.0, 12.9]	8.0 [5.0, 12.9]	7.6 [5.0, 13.2]	0.380
ICU length of stay, days	2.6 [1.7, 4.2]	2.6 [1.7, 4.1]	2.7 [1.7, 4.3]	0.495

HF: Heart failure; AF: Atrial fibrillation; CAD: Coronary artery disease; CKD: Chronic kidney disease; AKI: Acute kidney injury; WBC: White blood cell; Hb: Hemoglobin; PLT: Platelet; Cr: Creatinine; BUN: Blood urea nitrogen; T: Temperature; HR: Heart rate; RR: Respiratory rate; SBP: Systolic blood pressure; DBP: Diastolic blood pressure; SpO_2_: Peripheral oxygen saturation; MV: Mechanical ventilation; CRRT: Continuous renal replacement therapy; ICU: Intensive care unit

### Model construction and performance comparison

**Fig. 3:**
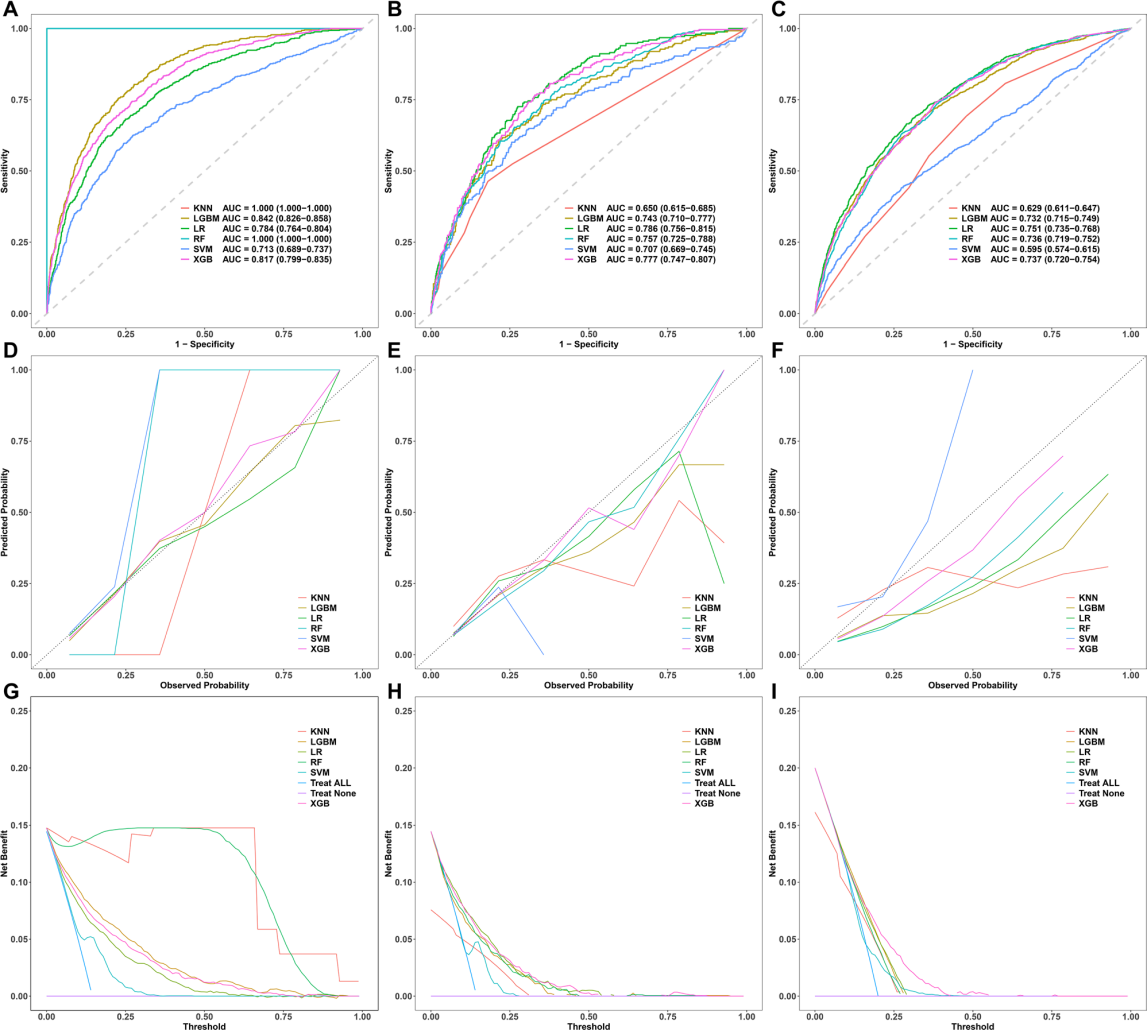
Performance comparison of models. (**A–C**) ROC curves. (**D–F**) Calibration plots. (**G–I**) Decision curves. ROC: Receiver operating characteristic; AUC: Area under the curve; KNN: K-nearest neighbor; LGBM: Light gradient boosting machine; LR: Logistic regression; RF: Random forest; SVM: Support vector machine; XGB: eXtreme gradient boosting

**Fig. 4:**
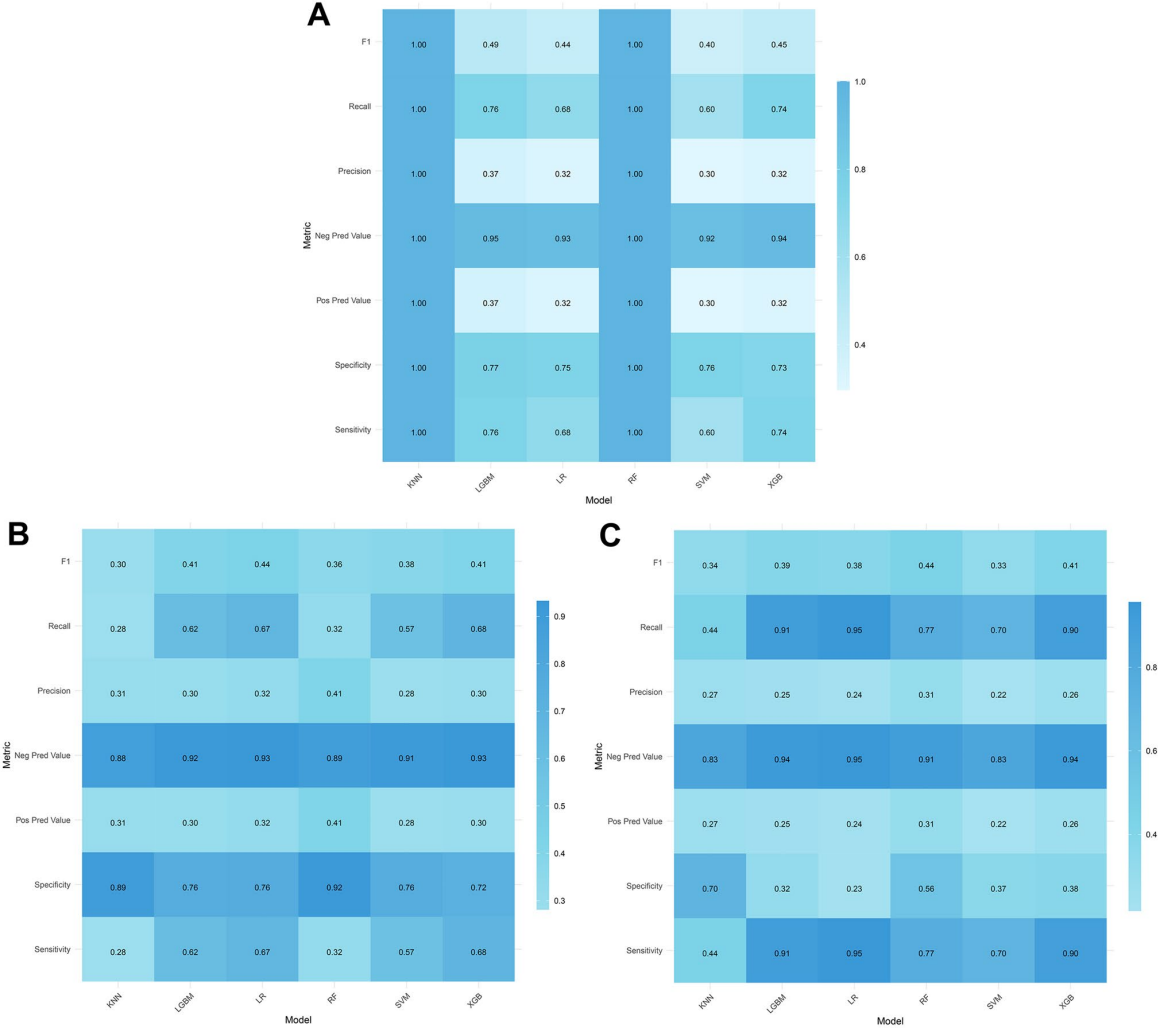
Confusion matrix for model classification performance index. (**A**) Training cohort. (**B**) Internal validation cohort. (**C**) External validation cohort. AUC: Area under the curve; KNN: K-nearest neighbor; LGBM: Light gradient boosting machine; LR: Logistic regression; RF: Random forest; SVM: Support vector machine; XGB: eXtreme gradient boosting

Twelve variables mentioned above were employed to construct six models (KNN, LGBM, LR, RF, SVM, and XGB) for predicting the in-hospital mortality in critically ill cancer patients. The discriminatory performance of each prediction model across the training cohort, internal validation cohort, and external validation cohort is presented in Figs. 3, 4 and Table 3. The selection of the optimal model necessitates a comprehensive consideration of the stability and generalization capabilities of key metrics, including the AUC, sensitivity, specificity, PPV, negative predictive value, and F1 score. The core clinical significance of developing these ML models lies in the accurate identification of patients at high risk of death, thereby enabling medical staff to secure crucial treatment time. In the context of ICU clinical practice, the primary objective of the model is to achieve high sensitivity, aiming to minimize the risk of overlooking high-risk patients. Although high sensitivity may be associated with a certain proportion of “false positives,” in the ICU setting, the consequences of such misclassifications are far less severe than the potential risks of missed diagnoses. Thus, models capable of attaining extremely high sensitivity are given precedence.

**Table 3:** Predictive performances of machine learning models for predicting in-hospital mortality

Class	Model	AUC (95%CI)	Sensitivity (recall)	Specificity	Pos pred value (precision)	Neg pred value	F1 score	Balanced accuracy
**Training cohort**								
	KNN	1.000 (1.000 − 1.000)	1.000	1.000	1.000	1.000	1.000	1.000
	LGBM	0.842 (0.826 − 0.858)	0.755	0.774	0.366	0.948	0.493	0.764
	LR	0.784 (0.764 − 0.804)	0.682	0.751	0.322	0.932	0.437	0.717
	RF	1.000 (1.000 − 1.000)	1.000	1.000	1.000	1.000	1.000	1.000
	SVM	0.713 (0.689 − 0.737)	0.597	0.756	0.298	0.916	0.397	0.677
	XGB	0.817 (0.799 − 0.835)	0.745	0.730	0.323	0.943	0.451	0.737
**Internal validation cohort**								
	KNN	0.650 (0.615 − 0.685)	0.282	0.894	0.311	0.881	0.296	0.588
	LGBM	0.743 (0.710 − 0.777)	0.621	0.759	0.303	0.922	0.407	0.690
	LR	0.786 (0.756 − 0.815)	0.673	0.762	0.324	0.932	0.437	0.718
	RF	0.757 (0.725 − 0.788)	0.323	0.923	0.415	0.890	0.363	0.623
	SVM	0.707 (0.669 − 0.745)	0.573	0.755	0.283	0.913	0.379	0.664
	XGB	0.777 (0.747 − 0.807)	0.685	0.724	0.296	0.932	0.413	0.705
**External validation cohort**								
	KNN	0.629 (0.611 − 0.647)	0.441	0.702	0.270	0.834	0.335	0.572
	LGBM	0.732 (0.715 − 0.749)	0.911	0.318	0.250	0.935	0.393	0.615
	LR	0.751 (0.735 − 0.768)	0.955	0.233	0.237	0.954	0.380	0.594
	RF	0.736 (0.719 − 0.752)	0.773	0.565	0.308	0.909	0.440	0.669
	SVM	0.595 (0.574 − 0.615)	0.704	0.369	0.218	0.833	0.333	0.536
	XGB	0.737 (0.720 − 0.754)	0.897	0.375	0.264	0.936	0.408	0.636

AUC: Area under the curve; CI: Confidence interval; KNN: K-nearest neighbor; LGBM: Light gradient boosting machine; LR: Logistic regression; RF: Random forest; SVM: Support vector machine; XGB: eXtreme gradient boosting

Both the KNN and RF models achieved an AUC of 1.000 in the training cohort. However, their performance deteriorated significantly in the internal and external validation cohorts, indicating substantial overfitting. In particular, these models showed markedly reduced performance stability and limited generalization ability in the external validation cohorts, evidenced by a sharp decline in sensitivity, rendering them unsuitable for the stability requirements of ICU applications.

The SVM model demonstrated the poorest performance across all datasets. In the external validation cohort, both the AUC (0.595) and sensitivity (0.704) were lower than those of other models, and the specificity further decreased to 0.369, suggesting its inadequate classification capabilities. The linear kernel or default parameters of SVM may be ineffective in capturing complex nonlinear relationships, especially in scenarios with imbalanced class distributions, rendering it unsuitable for high-risk prediction tasks in the ICU.

The LR model offers distinct advantages in terms of high sensitivity and model interpretability. In the external validation cohort, its sensitivity reached an impressive 0.955, indicating a robust ability to detect positive cases with minimal risk of missed diagnoses. This high sensitivity aligns with the primary goal of reducing missed diagnoses in the ICU. Additionally, the LR model exhibited a relatively high and stable AUC across the internal validation cohort (0.786) and external validation cohort (0.751), demonstrating good generalization properties. As a linear model, LR features a straightforward structure and transparent parameters, which is highly conducive to the interpretability of the decision-making process in clinical settings. Nonetheless, the LR model also has notable limitations. Its relatively low specificity of 0.233 implies a false positive rate of 76.7%, which may increase the burden of clinical secondary screening. The low PPV of 0.237 suggests a significant deficiency in the model’s ability to accurately classify positive cases. DCA results indicated that the net benefit of the LR model declined significantly in the high-threshold range (> 0.6), thereby restricting its practical clinical utility. Despite these drawbacks, the simplicity, interpretability, and high computational efficiency of the LR model make it suitable for initial applications in clinical scenarios where feature weights need to be clearly understood.

The LGBM model shares similarities with the LR model in terms of high sensitivity and robustness. In the external validation cohort, its sensitivity reached 0.911. However, it also suffers from low specificity (0.318) and PPV (0.250). The LGBM model demonstrated a more balanced performance in the external validation cohort, with balanced accuracy (0.615) and F1 score (0.393) surpassing those of the LR model (0.594 and 0.380, respectively). DCA results indicated that the LGBM model had an overall excellent performance, with strong threshold adaptability and good generalization ability. Nevertheless, the AUC values of the LGBM model in the internal (0.743) and external (0.732) validation cohorts were slightly lower than those of the LR model (0.786 and 0.751), suggesting a marginally weaker overall discriminatory power. As an algorithm based on the gradient-boosting framework, LGBM offers high computational efficiency and greater robustness in handling complex nonlinear data relationships, making it suitable for large-scale data processing and clinical implementation.

The XGB model excels in balancing sensitivity and specificity. In the external validation cohort, it demonstrated a sensitivity of 0.897 and a negative predictive value of 0.936, indicating its reliability in identifying high-risk patients and suitability for medical decision-making focused on minimizing missed diagnoses. In terms of model stability, the AUC of the XGB model exhibited relatively minor fluctuations across the training cohort (0.817), internal validation cohort (0.777), and external validation cohort (0.737), suggesting strong generalization ability. The DCA curve (external validation) further attests to its clinical utility, showing that within the threshold range of 0.3–0.6 (especially above 0.5), the net benefit of the XGB model significantly exceeded that of other models, highlighting its advantage in balancing the benefits of true positives and the costs of false positives. Although its specificity (0.375) and PPV (0.264) were relatively low, they were still superior to those of the LGBM and LR models, suggesting that the XGB model achieved a comparatively lower false positive rate.

In summary, the LR, LGBM, and XGB models all achieved extremely high sensitivity, fulfilling the core requirements of the ICU environment. The LR model exhibited an exceptional level of sensitivity (0.955 in the external validation cohort) and a commendable and stable AUC performance, effectively minimizing the risk of missed diagnoses for high-risk patients. Although the relatively high false positive rate exists, this can be mitigated through clinical monitoring. The reduction in missed diagnosis risk directly impacts patient survival rates. Moreover, the interpretability of the LR model is a significant advantage, enabling a straightforward analysis of the influence of each variable on the prediction outcome through feature coefficients, which is of particular importance in medical settings demanding transparent decision-making processes. The LGBM model’s performance metrics closely resemble those of the LR model. However, its “black-box” nature limits interpretability, making it challenging to trace the underlying prediction logic. This may pose a hurdle in medical scenarios with strict regulatory requirements, rendering the LR model a more favorable choice. The XGB model demonstrated the most well-balanced overall performance in the validation cohorts, accompanied by strong generalization ability. Compared to the LR model, the XGB model achieved a more optimal balance between sensitivity (0.897 in the external validation cohort) and specificity (0.375 in the external validation cohort), with slightly better false positive control. Additionally, it boasted the highest net benefit in DCA, making it suitable for decision-making scenarios that require a balance between clinical benefits and resource costs. Due to issues such as overfitting, poor stability, or subpar performance, the RF, KNN, and SVM models are not recommended as core models for this application.

Consequently, the LR and XGB models emerge as the optimal choices. Future research will concentrate on the features of the LR and XGB models to optimize the identification of high-risk patients while effectively controlling the false positive rate, ultimately providing a reliable risk-warning tool for ICU clinical practice.

### Nomogram

### Tools for clinical implementation

**Fig. 5:**
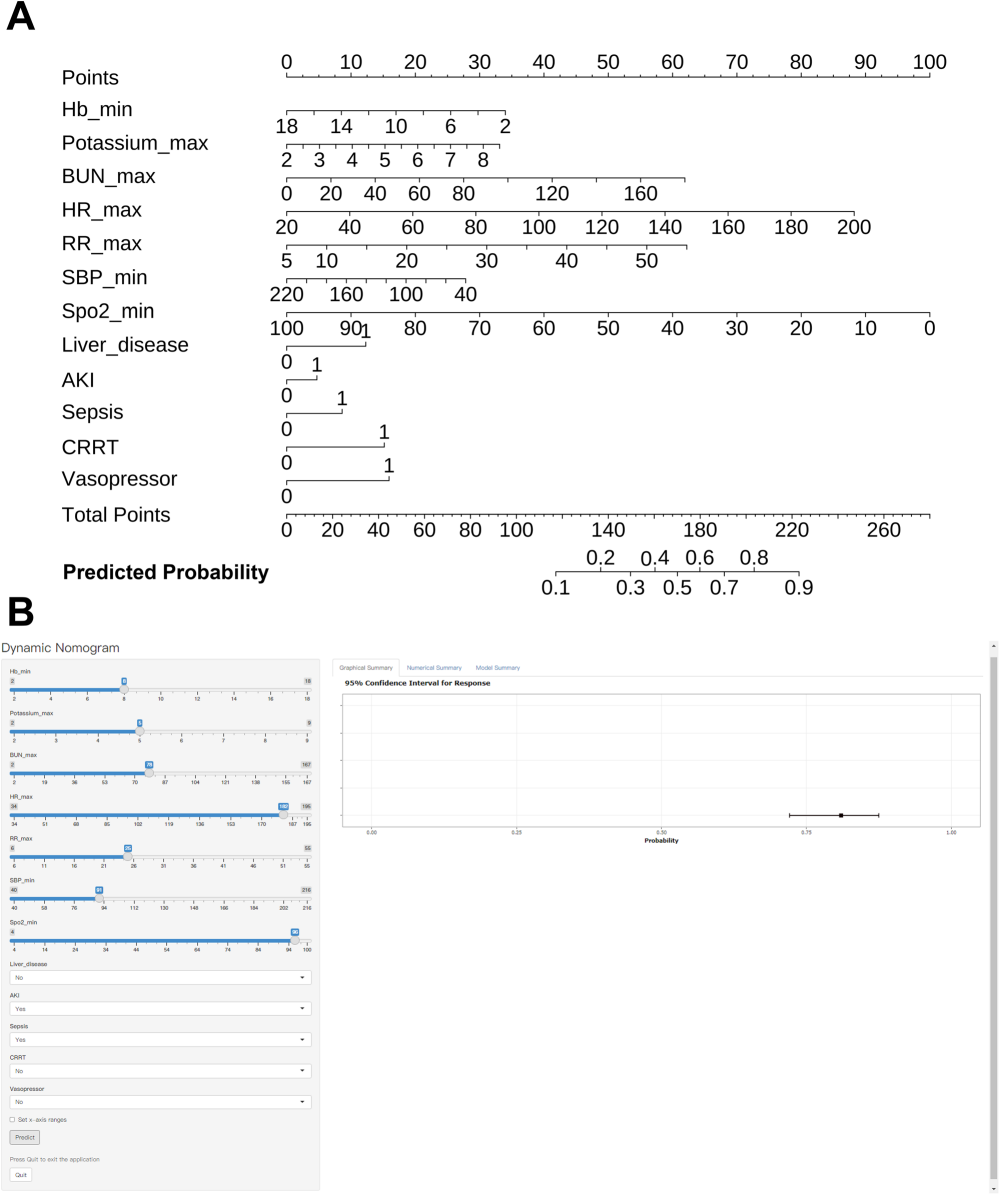
Nomograms for predicting the in-hospital mortality of cancer in ICU. (**A**) A static nomogram. (**B**) A dynamic online tool. ICU: Intensive care unit; Hb: Hemoglobin; BUN: Blood urea nitrogen; HR: Heart rate; RR: Heart rate; SBP: Systolic blood pressure; SpO_2_: Peripheral oxygen saturation; AKI: Acute kidney injury; CRRT: Continuous renal replacement therapy

Based on the LR model, nomograms for forecasting the in-hospital mortality in critically ill cancer patients were constructed (Fig. 5). Figure 5 presents a static nomogram. Although the nomogram offers convenience, precise values cannot be obtained during the calculation process. Hence, we established an online calculator based on the nomogram (https://icucancer2025.shinyapps.io/DynNomapp/) (Fig. 5) to streamline the computational procedure and enhance the accuracy of the predicted values.

### Threshold Selection for Clinical Implementation

The optimal cutoff values for the LR model in the training set, internal validation set, and external validation set were 0.153, 0.136, and 0.438, respectively. The corresponding sensitivity and specificity are presented in Table 3. The operational threshold of LR model was ultimately set at 0.153 through clinical calibration of model performance.

While external validation identified a Youden-optimal threshold of 0.438 (sensitivity = 66.5%, specificity = 71.0%) (Table 3), we prioritized sensitivity (95.5% at 0.153) over specificity (23.3%) to minimize missed diagnoses in clinical practice, as false positives could be resolved through subsequent confirmatory testing. This decision was validated by the threshold’s robust cross-population performance, maintaining sensitivity > 67% in training/internal validation sets while achieving 95.5% sensitivity in external validation.

### Model interpretability analysis based on SHAP

**Fig. 6:**
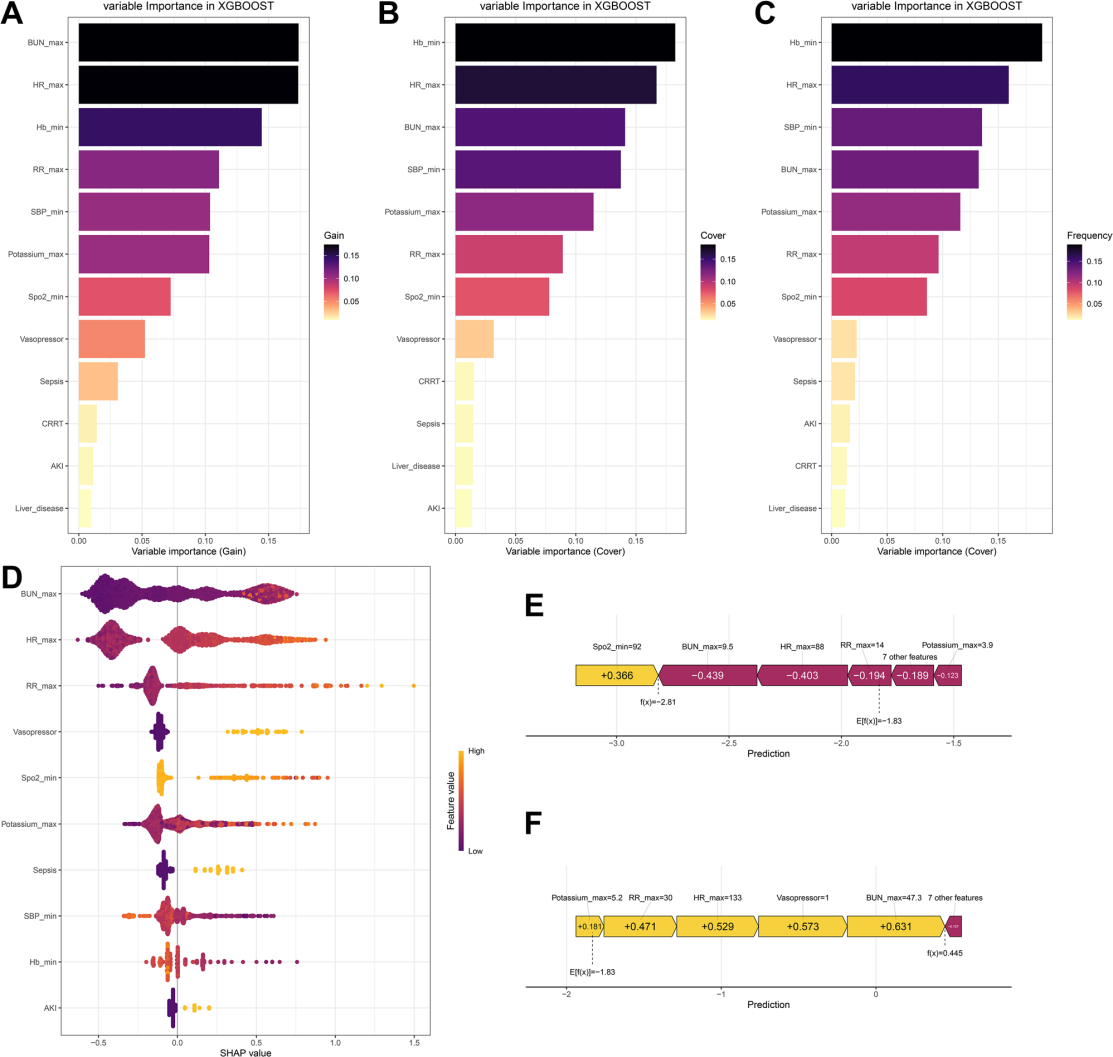
Explanation via SHAP method. (**A–C**) Variable importance plots. The analysis methods are “Gain”, “Cover”, and “Frequency”, respectively. (**D**) SHAP summary dot plot. (**E–F**) SHAP force plots for two representative survival (**E**) and dead (**F**) patients. BUN: Blood urea nitrogen; HR: Heart rate; Hb: Hemoglobin; RR: Heart rate; SBP: Systolic blood pressure; SpO_2_: Peripheral oxygen saturation; CRRT: Continuous renal replacement therapy; AKI: Acute kidney injury; SHAP: Shapley Additive exPlanations

In this study, the SHAP method was employed to explain the relative importance of various factors within the XGB model. This interpretable approach offers two levels of insights: a global overview at the feature level and detailed analysis for individual cases (Fig. 6).

### XGB model analysis at the global level

The variable importance plots (Fig. 6-C) show that BUN_max, HR_max, Hb_min, and SBP_min rank at the top in all three analysis approaches, namely “Gain”, “Cover”, and “Frequency”. This indicates that these variables hold global importance in the model’s decision-making. For example, BUN_max exhibits the highest importance in the Gain metric (0.55), suggesting that it makes a substantial contribution to enhancing the predictive power of the model.

The SHAP summary dot plot (Fig. 6) lists the top 10 most significant variables in order of importance and visually elucidates the direction and magnitude of their influence on model predictions. It can be observed from the figure that among the conventional indicators, BUN_max, HR_max, RR_max, Vasopressor, and SpO_2__min are the top five potent predictive variables.

Furthermore, higher levels of SpO_2__min, SBP_min, Hb_min were negatively associated with in-hospital mortality. In contrast, higher levels of BUN_max, HR_max, RR_max, Potassium_max, the utilization of vasopressor, the incidence of sepsis, and AKI were positively associated with in-hospital mortality.

### XGB model analysis at the individual level

To illustrate the positive or negative relationship between significant variables and in-hospital mortality, SHAP values were utilized to identify risk factors for death. A representative surviving patient and a deceased patient were respectively selected for detailed interpretability analysis (Fig. 6-F).

For the surviving patient (Fig. 6), the predicted probability of in-hospital mortality was relatively low. Specifically, with BUN_max = 9.5 mmol/L (contributed a SHAP value of - 0.439), HR_max = 88 bpm (contributed - 0.403), RR_max = 14 bpm (contributed - 0.194), and Potassium_max = 3.9 mmol/L (contributed - 0.123).

For the death patient (Fig. 6), the predicted probability of in-hospital mortality was relatively high. Specifically, with BUN_max = 47.3 mmol/L (contributed + 0.631), HR_max = 133 bpm (contributed + 0.529), RR_max = 30 bpm (contributed + 0.471), Potassium_max = 5.2 mmol/L (contributed + 0.181), the utilization of vasopressor (contributed + 0.573).

By visualizing the SHAP values of these samples, we are able to discern the influence of each feature on the model’s predictions for these specific cases.

## Discussion

The proportion of cancer patients requiring ICU admission is substantial, necessitating focused attention and timely intervention [2–4]. This study successfully established a prediction model for the in-hospital mortality in critically ill cancer patients and further substantiated its generalizability through external validation.

For a considerable period, generalized linear models (such as LR regression) have served as significant tools for examining the relationship between target outcomes and associated risk factors [19]. Recently, the exploration of ML models in artificial intelligence has garnered escalating attention. This methodology is inherently proficient in conducting automatic analyses of complex datasets. In contrast to traditional statistical analysis approaches, ML models exhibit notably superior performance, require less time, and possess higher accuracy, significantly enhancing work efficiency [20]. Ko et al. constructed the ML model CanICU for predicting the 28-day mortality rate of adult cancer patients in the ICU using nine variables [21]. Danilatou et al. developed ML models for predicting the early and late mortality rates of cancer patients in the ICU, with AUCs of 0.94 and 0.74–0.88, respectively, outperforming traditional scoring systems [22]. Bu et al. developed an ML model for evaluating the in-hospital mortality of cancer patients and hyperkalemia, among which the XGB model was optimal, attaining the highest AUC of 0.733 in the validation cohort [13].

ML techniques are often characterized as “black boxes” as most studies fail to explain how the key clinical variables and prediction results derived from ML techniques are obtained. This may lead clinicians to refrain from their utilization as they are unwilling to make medical decisions based on unclear or non-transparent information. In this research, we utilized the SHAP method to demystify the “black box” characteristics of ML models. By visualizing critical clinical variables, the SHAP method provides insights into how these models make predictions. Furthermore, by inputting specific cases, it is possible to obtain specific predicted outcomes for those cases, explaining ML models at an individual level. In comparison with ML models, LR models not only offer visualizations of key clinical variables but also generate nomograms and web calculators to assist physicians in clinical decision-making and timely intervention. In this study, we respectively utilized LR and five ML models (KNN, LGBM, RF, SVM, XGB) for the early prediction of the target outcome. AUC is a comprehensive metric for evaluating model performance. Since our objective is to identify patients at risk of in-hospital mortality early, Sensitivity is also an important evaluation metric. Consistent with the aforementioned statements, our research findings affirm the superiority of the XGB model. The XGB model demonstrated outstanding predictive potential in forecasting the in-hospital mortality in critically ill cancer patients. This reinforces the prominent position of XGB among a series of ML methods.

A vast array of clinical variables can be acquired in the ICU, particularly in this study, where there are as many as sixty-one clinical variables. Nevertheless, risk variables often exhibit correlations. Owing to potential issues such as overfitting and multicollinearity, this poses challenges to traditional statistical methods. Hence, prior to model construction in this study, the LASSO regression method was utilized for feature selection. LASSO regression facilitates the creation of a more succinct model by retaining only the most influential predictors, thereby enhancing the model’s interpretability and generalizability.

The significant findings of this study reveal that BUN, potassium, Hb, HR, SBP, vasopressor, RR, SpO_2_ are crucial predictors of in-hospital mortality in critically ill cancer patients.

The BUN level not only reflects the excretory function of the kidneys but also indirectly indicates the metabolic burden and tissue perfusion status of cancer patients [23]. Research indicates that BUN at ICU admission is closely associated with mortality, and this risk is independent of creatinine and other risk factors [24]. The 30-day mortality rate of ICU patients with BUN > 40 mg/dL is 1.5 times higher than that of the group with BUN 10–20 mg/dL (OR = 1.53, 95% CI 1.40–1.68). This association might be intimately related to the protein catabolic metabolism and net negative nitrogen balance in cancer patients [25]. Additionally, the persistent hypercatabolic state in critically ill patients further leads to a decline in immune function.

A serum potassium level within the range of 3.5–5.0 mmol/L is regarded as the clinically safe threshold. Significantly, electrolyte imbalance has been shown to have a strong association with the risk of malignant arrhythmias and sudden cardiac death in critically ill patients. Existing investigations have established a non-linear relationship (– U - shaped curve) between the mortality rate of ICU patients and serum potassium levels [26]. Hyperkalemia, defined as a serum potassium level greater than 5.5 mmol/L, can be induced by multiple factors in the population of cancer patients. These factors include AKI, tumor lysis syndrome, or medications. From an electrophysiological perspective, hyperkalemia can precipitate life-threatening ventricular arrhythmias by altering the resting potential of myocardial cells. Given that cancer patients are frequently exposed to nephrotoxic therapies and the risk of tumor lysis syndrome, the incidence of electrolyte disorders among them is substantially higher than that in the general population. Although CRRT has been identified as the most effective intervention for hyperkalemia, critically ill cancer patients often exhibit reduced tolerance to this treatment due to hemodynamic instability.

Epidemiological research indicates that the prevalence of anemia among cancer patients is as high as 39%. However, only 40% of these cases receive treatment [27]. In cancer patients, a reduction in Hb levels can be attributed to various factors, such as bone marrow suppression, chronic inflammatory states, occult blood loss, or nutritional deficiencies.

The complex interplay between tumor cells and the immune system can also give rise to anemia. The overexpression of certain inflammatory cytokines may further contribute to the development of anemia in these patients [28]. In the ICU setting, iatrogenic factors, such as hemodilution resulting from fluid resuscitation and frequent diagnostic blood sampling, exacerbate the decline in Hb levels. Evidence from studies has shown a significant association between low Hb levels and mortality within six months following hip fracture surgery [29].

Tachycardia, often considered a compensatory circulatory response, is commonly incorporated into ICU prognosis assessment models [30]. However, its clinical implications are more complex in cancer patients. Tachycardia can be triggered by a variety of factors, including infection, sympathetic overactivity, and hypovolemia. Persistent tachycardia increases myocardial oxygen consumption and reduces the diastolic filling time of the heart, leading to a decrease in cardiac output. This effect is particularly pronounced in patients with coronary artery disease or chemotherapy-related cardiomyopathy, where it may even precipitate heart failure. Tumors themselves or paraneoplastic syndromes can directly stimulate sympathetic overactivity. Some studies have suggested a U-shaped relationship between the 28-day mortality rate of sepsis patients and the HR_max. The optimal prognosis is observed when HRmax is maintained within the range of 70–110 bpm [31]. Furthermore, in patients with septic shock, controlling the HR to 85 bpm or lower can significantly enhance in-hospital survival rates [32]. This finding underscores the crucial role of HR regulation in the management of critically ill patients [33]. It is important to note that due to their immunocompromised state and limited organ function reserve, cancer patients have a significantly lower tolerance threshold for circulatory decompensation. As a result, they are more susceptible to progressing to circulatory failure.

Fluctuations in sbp often mirror the critical state of circulatory failure and organ hypoperfusion [34]. The underlying pathology is closely associated with advanced tumors complicated by septic shock. Tumor-specific factors, such as adrenal insufficiency resulting from adrenal metastases and anti-angiogenic drugs, can exacerbate hypotension. Hypotension typically necessitates the use of vasoactive medications. However, these drugs may further exacerbate arrhythmias or visceral ischemia, thereby establishing a vicious cycle. This study also highlights the significant predictive value of vasoactive drugs for in-hospital mortality. The use of vasoactive drugs, such as norepinephrine, serves as a strong indicator of the severity of the patient’s condition, often suggesting the presence of refractory shock. Moreover, a positive correlation exists between the dosage of vasoactive drugs and mortality [35], indicating that these drugs not only represent a therapeutic intervention but also serve as a marker of disease severity.

An elevated RR and decreased SpO₂ are compensatory responses to hypoxemia. Cancer patients frequently have multiple comorbidities, such as chronic lung diseases. Given their limited pulmonary function reserve and restricted respiratory compensatory capacity, these patients are highly vulnerable to rapid progression to respiratory failure upon the onset of pulmonary infections, pleural effusions, or acute respiratory distress syndrome. Ventilation insufficiency resulting from respiratory muscle fatigue can further exacerbate hypercapnia and hypoxemia, ultimately increasing the need for MV.

One of the key strengths of this study lies in the retrospective analysis of two large public databases encompassing extensive information on critically ill patients and the division of these data into clinically meaningful sets (demographic characteristics, comorbidities, laboratory indicators, vital signs, and intervention measures). Secondly, in contrast to the majority of previous studies that merely considered static laboratory indicator variables, this study also took into account dynamic factors, collecting the maximum and minimum values of laboratory indicator variables. The variables included in this study also encompassed intervention measures, enabling a multi-faceted exploration of factors influencing disease progression. Additionally, the variables utilized in the study model are all readily measurable and obtainable clinical indicators with high clinical application value, facilitating the model’s promotion in hospitals at all levels. Finally, we also constructed a web calculator to aid clinicians in the early identification of high-risk individuals and meet the requirements of clinical decision-making.

In interpreting the findings of this study, it is important to consider several limitations. Firstly, this study is of a retrospective nature, with data derived from hospital medical records, inevitably entailing selection bias. There are obvious differences in the baseline characteristics of the two databases. In the internal validation, failure to isolate data according to centers may introduce biases. However, the models of this study were externally validated in another database and performed well, demonstrating the robustness of the model’s predictive performance. In the future, data from additional centers should be incorporated, with strict center-wise validation methods applied. This will help further verify the model’s robustness. Secondly, this study only included clinical indicators common to all databases with a missing rate below 20%. Potentially important indicators not included, such as albumin, may also substantially impact in-hospital mortality. Thirdly, the present study primarily focuses on interpretable ML models but does not incorporate emerging deep learning techniques such as Transformers or Convolutional Neural Networks. In future work, we aim to integrate deep learning approaches and investigate automatic feature engineering methods to further improve model performance. Finally, the present study utilized data from two large public U.S. critical care databases, which may constrain the model’s generalizability owing to high data homogeneity. In the future, data from more regions and healthcare systems should be included. This should cover various types of ICUs, such as general ICUs and oncology-specific ICUs, to further validate and optimize the model.

## Conclusions

In summary, this study constructed and validated a prediction model for in-hospital mortality in critically ill cancer patients, among which the LR model and the XGB model exhibited the best efficacy. Additionally, twelve predictors (Hb_min, Potassium_max, BUN_max, HR_max, RR_max, SBP_min, SpO_2__min, Liver_disease, AKI, Sepsis, CRRT, Vasopressor) were determined, which can predict the adverse outcomes of patients with relatively high accuracy. Furthermore, this study offered nomogram and web calculator as convenient and rapid assessment tools, facilitating clinicians to promptly identify patients at high risk of in-hospital mortality, adjust treatment and management plans in a timely manner, and thereby improve the prognosis of these patients.

## Electronic supplementary material

Below is the link to the electronic supplementary material.

Supplementary Material 1

## Data Availability

The original contributions presented in the study are publicly available. These databases can be found here: [https://eicu-crd.mit.edu/gettingstarted/access/]& [https://physionet.org/content/mimiciv/3.0/].

## Abbreviations

AKI: Acute kidney injury
AUC: Area under the curve
BUN: Blood urea nitrogen
CI: Confidence interval
CRRT: Continuous renal replacement therapy
DCA: Decision curve analysis
Hb: Hemoglobin
HR: Heart rate
ICD-9: International Classification of Diseases, Ninth Revision
ICD-10: International Classification of Diseases, Tenth Revision
ICU: Intensive care unit
KNN: K-nearest neighbor
LASSO: Least absolute shrinkage and selection operator
LGBM: Light gradient boosting machine
LR: Logistic regression
ML: Machine learning
MV: Mechanical ventilation
PPV: Positive predictive value
RF: Random Forest
ROC: Receiver operating characteristic
RR: Respiratory rate
SBP: Systolic blood pressure
SHAP: Shapley Additive exPlanations
SpO_2_: Peripheral oxygen saturation
SVM: Support vector machine
XGB: eXtreme gradient boosting
