# Supplementary material for: Machine learning-based predictive tools and nomogram for in-hospital mortality in critically ill cancer patients: development and external validation using retrospective cohorts
Source: BMC Med Inform Decis Mak. 2025 Jul 4;25:251. doi: 10.1186/s12911-025-03054-z (PMC12231625; doi:10.1186/s12911-025-03054-z)
Supplement: Supplementary file 1 — Supplementary Material 1 [file 12911_2025_3054_MOESM1_ESM.docx]

**Table S1. List of ICD-9/ICD-10 codes to identify each cancer.**

| **Type** | **Category** | **Codes** |
| --- | --- | --- |
| ICD-9 | Head and neck | 140–149.9, 160*, 161*, 162*, 195.0 |
|  | Non-colon GI | 150–152.9, 155–159.9, 235*, 239.0 |
|  | Colon | 153*, 154*, 235.2 |
|  | Lung | 162*, 235.9, 239.1 |
|  | Pleura/mediastinum | 163*, 164* |
|  | Bones/soft tissue | 170*, 171*, 238.1, 238.2 |
|  | Melanoma | 172* |
|  | Non-melanomatous skin cancer | 173*, 238.2 |
|  | Breast | 174*, 175*, 239.3 |
|  | Gyn | 180*, 182*, 183*, 184*, 236.1, 236.2 |
|  | Prostate | 185*, 236.5 |
|  | Testes/Male GU | 186*, 187.3, 187.4, 187.9, 236.4, 236.6 |
|  | Urinary Tract | 188*, 189*, 236.7, 236.91, 239.4, 239.5 |
|  | Brain | 190–192.9, 237.5, 237.6, 239.6 |
|  | Endocrine | 193*, 194*, 237.0, 237.4, 239.7 |
|  | Non-specific site | 195*, 199*, 238.8, 238.9, 239.8, 239.9 |
|  | Lymph node spread | 196* |
|  | Lymphatic, hematopoietic, and related tissues | 200*, 201*, 202*, 203*, 204* |
| ICD-10 | Malignant neoplasms of lip, oral cavity and pharynx | C00–C14 |
|  | Malignant neoplasms of digestive organs | C15–C26 |
|  | Malignant neoplasms of respiratory and intrathoracic organs | C30–C39 |
|  | Malignant neoplasms of bone and articular cartilage | C40–C41 |
|  | Melanoma and other malignant neoplasms of skin | C43–C44 |
|  | Malignant neoplasms of mesothelial and soft tissue | C45–C49 |
|  | Malignant neoplasm of breast | C50–C50 |
|  | Malignant neoplasms of female genital organs | C51–C58 |
|  | Malignant neoplasms of male genital organs | C60–C63 |
|  | Malignant neoplasms of urinary tract | C64–C68 |
|  | Malignant neoplasms of eye, brain and other parts of central nervous system | C69–C72 |
|  | Malignant neoplasms of thyroid and other endocrine glands | C73–C75 |
|  | Malignant neoplasms of unclear, secondary, and unspecified location | C76-C80 |
|  | Malignant neoplasms of lymphatic, hematopoietic, and related tissues | C81-C96 |
|  | Non-specific site | C97 |

**Table S2. Missing data of variables.**

| **Variables** | **eICU database** | | | **MIMIC database** | | |
| --- | --- | --- | --- | --- | --- | --- |
|  | **Available quantity** | **Missing quantity** | **Missing value percentage** | **Available quantity** | **Missing quantity** | **Missing value percentage** |
| Age | 5718 | 0 | 0.0 | 5110 | 0 | 0.0 |
| Gender | 5718 | 0 | 0.0 | 5110 | 0 | 0.0 |
| BMI | 5626 | 92 | 1.6 | 2191 | 2919 | 57.1 |
| Hypertension | 5718 | 0 | 0.0 | 5110 | 0 | 0.0 |
| Diabetes | 5718 | 0 | 0.0 | 5110 | 0 | 0.0 |
| Liver disease | 5718 | 0 | 0.0 | 5110 | 0 | 0.0 |
| Peripheral vascular disease | 5718 | 0 | 0.0 | 5110 | 0 | 0.0 |
| Cerebrovascular disease | 5718 | 0 | 0.0 | 5110 | 0 | 0.0 |
| HF | 5718 | 0 | 0.0 | 5110 | 0 | 0.0 |
| AF | 5718 | 0 | 0.0 | 5110 | 0 | 0.0 |
| CAD | 5718 | 0 | 0.0 | 5110 | 0 | 0.0 |
| CKD | 5718 | 0 | 0.0 | 5110 | 0 | 0.0 |
| AKI | 5718 | 0 | 0.0 | 5110 | 0 | 0.0 |
| Sepsis | 5718 | 0 | 0.0 | 5110 | 0 | 0.0 |
| WBC_max | 5530 | 188 | 3.3 | 5076 | 34 | 0.7 |
| Hb_min | 5558 | 160 | 2.8 | 5078 | 32 | 0.6 |
| PLT_min | 5475 | 243 | 4.2 | 5076 | 34 | 0.7 |
| Alb_min | 3279 | 2439 | 42.7 | 1573 | 3537 | 69.2 |
| Potassium_max | 5591 | 127 | 2.2 | 4978 | 132 | 2.6 |
| Sodium_min | 5594 | 124 | 2.2 | 4978 | 132 | 2.6 |
| Chloride_max | 5572 | 146 | 2.6 | 4977 | 133 | 2.6 |
| Calcium_min | 5445 | 273 | 4.8 | 4961 | 149 | 2.9 |
| BG_max | 5562 | 156 | 2.7 | 5099 | 11 | 0.2 |
| ALT_max | 3069 | 2649 | 46.3 | 2414 | 2696 | 52.8 |
| AST_max | 3096 | 2622 | 45.9 | 2429 | 2681 | 52.5 |
| ALP_max | 3072 | 2646 | 46.3 | 2406 | 2704 | 52.9 |
| TBil_max | 3063 | 2655 | 46.4 | 2411 | 2699 | 52.8 |
| Cr_max | 5592 | 126 | 2.2 | 5103 | 7 | 0.1 |
| BUN_max | 5587 | 131 | 2.3 | 5104 | 6 | 0.1 |
| AG_max | 4438 | 1280 | 22.4 | 5106 | 4 | 0.1 |
| Bicarbonate_min | 5285 | 433 | 7.6 | 5107 | 3 | 0.1 |
| INR_max | 2687 | 3031 | 53.0 | 4255 | 855 | 16.7 |
| PT_max | 2586 | 3132 | 54.8 | 4255 | 855 | 16.7 |
| PTT_max | 1905 | 3813 | 66.7 | 4213 | 897 | 17.6 |
| T_max | 5556 | 162 | 2.8 | 4552 | 558 | 10.9 |
| HR_max | 5400 | 318 | 5.6 | 4500 | 610 | 11.9 |
| RR_max | 5185 | 533 | 9.3 | 4480 | 630 | 12.3 |
| SBP_min | 5367 | 351 | 6.1 | 4278 | 832 | 16.3 |
| DBP_min | 5367 | 351 | 6.1 | 4270 | 840 | 16.4 |
| Spo2_min | 4824 | 894 | 15.6 | 4645 | 465 | 9.1 |
| MV | 5112 | 0 | 0.0 | 5110 | 0 | 0.0 |
| CRRT | 5718 | 0 | 0.0 | 5110 | 0 | 0.0 |
| Vasopressor | 5718 | 0 | 0.0 | 5110 | 0 | 0.0 |
| Antibiotic | 5718 | 0 | 0.0 | 5110 | 0 | 0.0 |
| Antiarrhythmic | 5718 | 0 | 0.0 | 5110 | 0 | 0.0 |
| Blood product | 5718 | 0 | 0.0 | 5110 | 0 | 0.0 |
| Diuretic | 5718 | 0 | 0.0 | 5110 | 0 | 0.0 |
| Sedative | 5718 | 0 | 0.0 | 5110 | 0 | 0.0 |

BMI: Body mass index; HF: Heart failure; AF: Atrial fibrillation; CAD: Coronary artery disease; CKD: Chronic kidney disease; AKI: Acute kidney injury; WBC: White blood cell; Hb: Hemoglobin; PLT: Platelet; Alb: Albumin; BG: Blood glucose; ALT: Alanine aminotransferase; AST: Aspartate transaminase; ALP: Alkaline phosphatase; TBil: Total bilirubin; Cr: Creatinine; BUN: Blood urea nitrogen; AG: Anion gap; INR: International normalized ratio; PT: Prothrombin time; PTT: Partial thromboplastin time; T: Temperature; HR: Heart rate; RR: Respiratory rate; SBP: Systolic blood pressure; DBP: Diastolic blood pressure; Spo2: Peripheral oxygen saturation; MV: Mechanical ventilation; CRRT: Continuous renal replacement therapy.

**Table S3. Correspondence between lambda values and selected variables.**

| **Log Lambda** | **Lambda value** | **Model degrees of freedom (number of variables)** | **Selected variables (non-zero coefficients)** | **Coefficients** |
| --- | --- | --- | --- | --- |
| -6.23 | 0.001973207 | 30 | CRRT | 0.853209549 |
|  |  |  | Cerebrovascular disease | 0.682637635 |
|  |  |  | Vasopressor | 0.611163014 |
|  |  |  | Liver disease | 0.442367662 |
|  |  |  | Sepsis | 0.436779533 |
|  |  |  | AKI | 0.247838116 |
|  |  |  | Potassium_max | 0.224818813 |
|  |  |  | AF | 0.220745887 |
|  |  |  | Sedative | 0.212598480 |
|  |  |  | Diuretic | 0.099061843 |
|  |  |  | MV | 0.077880123 |
|  |  |  | Sodium_min | 0.064117576 |
|  |  |  | RR_max | 0.046953634 |
|  |  |  | HR_max | 0.020290618 |
|  |  |  | BUN_max | 0.019659945 |
|  |  |  | WBC_max | 0.004144996 |
|  |  |  | Age | 0.003894547 |
|  |  |  | Glucose_max | -0.000712418 |
|  |  |  | SBP_min | -0.006335827 |
|  |  |  | Blood product | -0.008959926 |
|  |  |  | Spo2_min | -0.038608378 |
|  |  |  | Bicarbonate_min | -0.038840177 |
|  |  |  | Chloride_max | -0.060423934 |
|  |  |  | Antiarrhythmic | -0.071428307 |
|  |  |  | Hb_min | -0.095130186 |
|  |  |  | Gender | -0.110858290 |
|  |  |  | HF | -0.119567601 |
|  |  |  | CKD | -0.139581834 |
|  |  |  | T_max | -0.194766190 |
|  |  |  | Cr_max | -0.225769231 |
| -4.09 | 0.016767400 | 12 | Vasopressor | 0.526526503 |
|  |  |  | CRRT | 0.314741929 |
|  |  |  | Sepsis | 0.278201469 |
|  |  |  | Liver disease | 0.196357473 |
|  |  |  | AKI | 0.128798859 |
|  |  |  | RR_max | 0.039368714 |
|  |  |  | HR_max | 0.016094529 |
|  |  |  | Potassium_max | 0.014566549 |
|  |  |  | BUN_max | 0.014379343 |
|  |  |  | SBP_min | -0.002084105 |
|  |  |  | Spo2_min | -0.022676096 |
|  |  |  | Hb_min | -0.023119830 |

CRRT: Continuous renal replacement therapy; AKI: Acute kidney injury; AF: Atrial fibrillation; MV: Mechanical ventilation; RR: Respiratory rate; HR: Heart rate; BUN: Blood urea nitrogen; WBC: White blood cell; SBP: Systolic blood pressure; Spo2: Peripheral oxygen saturation; Hb: Hemoglobin; HF: Heart failure; CKD: Chronic kidney disease; T: Temperature; Cr: Creatinine.
